# The correlation between anisogamy and sexual selection intensity—the broad theoretical predictions

**DOI:** 10.1093/evlett/qrae029

**Published:** 2024-07-12

**Authors:** Jussi Lehtonen, Geoff A Parker

**Affiliations:** Department of Biological and Environmental Science, University of Jyväskylä, Jyväskylä, Finland; Department of Evolution, Ecology and Behaviour, University of Liverpool, Liverpool, United Kingdom

**Keywords:** anisogamy, behavior, sexual selection, theory

## Abstract

Darwin and Bateman suggested that precopulatory sexual selection is more intense on males than females, and that this difference is due to anisogamy (i.e., dimorphism in gamete size and number). While a recent paper apparently presents empirical support for this hypothesis, another appears at first sight to present evidence against it. We argue that this is partly due to lack of transparent theoretical predictions, and discuss and analyze sexual selection theory in relation to anisogamy evolution. On one hand, we find that there exists relatively little theory that can directly address all the tested predictions; on the other, the picture painted by current theory indicates that both sets of empirical results broadly match predictions about the causal link between anisogamy and sexual selection, thus reconciling the two apparently opposing claims. We also discuss in a very broad, general sense how anisogamy is expected to affect sexual selection.

Why do the two sexes have a mainstream tendency to evolve in different directions, creating asymmetric “sex roles,” particularly in terms of reproductive traits? While the question dates back to the writings of Darwin (1871), Bateman (1948) later explicitly claimed that the difference in the strength of sexual selection is ultimately caused by the difference in gamete number: males make more gametes than females, typically by a very large margin (e.g., Lüpold & Fitzpatrick, 2015; Parker, 1982). Though this question has since been investigated by many others, it remains controversial and debated.

Following Darwin and Bateman’s claims, most measures of “the intensity of sexual selection” relate to preejaculatory sexual selection (competition for matings and mate choice), which is our main focus here, rather than postejaculatory sexual selection (competition among gametes for fertilizations and mate choice by gamete selection). Our article is motivated by what we see as a gap in the theoretical literature and in its interpretation, and by two important recent analyses of empirical evidence with seemingly contradictory outcomes. Defining the two sexes by their gamete size, Janicke et al. (2016) show that precopulatory sexual selection is stronger in males than in females across the animal kingdom—in other words, in anisogamous organisms, the strength of sexual selection tends to be stronger in the type making smaller gametes. Mokos et al. (2021), however, found no relationship between the degree of gamete size difference (anisogamy) and the intensity of sexual selection. The former is described as a confirmation of the “Darwin–Bateman paradigm” while the latter is claimed to question it (see also Szekely, 2023). Here we clarify the relevant theoretical predictions and explain why there is no conflict between these results when considered through the lens of current theoretical understanding.

How can we characterize the nature of the theory being tested by Janicke et al. (2016) and Mokos et al. (2021)? Firstly, the conclusion of Mokos et al. is predicated on the idea that a detectable relationship between the degree of anisogamy (in the range found in metazoans) and the intensity of sexual selection is implied by sexual selection theory. Conversely, if theory does not entail such a prediction, the results of Mokos et al. do not contradict the Darwin–Bateman paradigm. Second, these empirical tests are quantitative in nature, and thus should ideally be founded in mathematical, evolutionary models rather than verbal predictions. Note that the majority of published mathematical theory in precopulatory sexual selection is largely irrelevant to this question: a recent survey of sexual selection theory by de Vries and Lehtonen (2023) found that most theoretical models in precopulatory sexual selection neither explicitly define the sexes in the first place, nor provide any causal link from gamete dimorphism to sexual selection. This does not imply that these models are wrong, but it does mean they are silent regarding the nature of the correlation between anisogamy and sexual selection. Evron (2023) similarly observed that many models in sexual selection do not make use of definitional properties of the sexes. However, these two studies came to very different conclusions and recommendations: de Vries and Lehtonen advocated development of new theory to stand alongside and complement existing work, while Evron suggested that sexual selection theory should dispense with the notion of the sexes altogether. We take the former position.

While more modeling work regarding the relationship between anisogamy and sexual selection is needed (de Vries & Lehtonen, 2023), a natural question is: where do the theoretical predictions underlying the studies of Janicke et al. (2016) and Mokos et al. (2021) come from, if most models cannot address them? If they do arise from existing mathematical theory of precopulatory sexual selection, they can only come from a small subset of such theory where gamete size is explicitly included. Here, we examine two types of models that explicitly address sexual selection and gamete size. One analyses directly the causal link from gamete size to precopulatory traits as the gametic system transitions from isogamy to anisogamy. The other type of model we examine is concerned with the evolution of gamete size when strong anisogamy has already evolved, and the intensity of pre- and postcopulatory sexual selection vary. Both could, at least in principle, predict a correlation between the anisogamy ratio and the intensity of precopulatory sexual selection.

We find that an overall result of these types of models is that the strength of sexual selection should indeed be correlated with the extent of anisogamy under weak anisogamy ratios. However, under strong anisogamy (typical across Metazoa), there is no obvious prediction of a sustained correlation between the anisogamy ratio and the intensity of sexual selection. The overall prediction across multicellular animals, then, is a positive but near-binary correlation between anisogamy and precopulatory sexual selection, and this prediction is fully compatible with the results of both Janicke et al. (2016) and Mokos et al. (2021)—thus the results of both articles provide valuable and consistent empirical evidence on the nature of sexual selection.

To reiterate, the two claims we wish to analyze are as below:

**Claim 1**) A difference in gamete size and number leads to different evolutionary trajectories between the sexes, with males generally experiencing more intense sexual selection. Janicke et al. (2016) test this claim and find support for it.
**Claim 2**) The degree of gamete size difference should be correlated with the degree of sexual selection across typically observed gamete size ratios. Mokos et al. (2021) test this claim and do not find support for it.

Claim 1 was initially articulated by Bateman (1948) and has been repeatedly emphasized as a central aspect of the “sexual cascade” (Parker, 2014) and in theoretical models (e.g., Lehtonen et al., 2016b). However, we are not aware of theory that predicts a sustained correlation between anisogamy and sexual selection, as in Claim 2.

The underlying reasoning for Claim 1 is simple: Once one gamete type outnumbers the other, with a unity adult sex ratio only one sex can in principle have all its gametes fuse with a gamete of opposite sex in a single mating, while the other will have surplus gametes, enabling resources to be reallocated into precopulatory sexual competition (Lehtonen et al., 2016b; Parker, 2014). In the simplest scenarios, this is a binary condition (all gametes can or cannot be fused in a single mating), and we explain below in more detail why it alters selection on the sexes.

Why the correlation of Claim 2 is likely undetectable in nature: A simple reason is that the great majority of anisogamous organisms have a vast difference in the size and number of female and male gametes (Lüpold & Fitzpatrick, 2015; Parker, 1982), that is, female:male gamete size and number ratios are typically many orders of magnitude different from unity. The binary condition of Claim 1 is fulfilled as gametes begin to diverge, and according to recent models spanning the isogamy-anisogamy continuum (Lehtonen, 2022; Lehtonen et al., 2016b) the system “saturates” relatively quickly, so that further divergence in gamete size and number does not directly cause further divergence in sexual selection. In other words, the anisogamy ratio in most multicellular animals is so high that we should not expect a sustained correlation between anisogamy ratio and sexual selection. Only in the highly aberrant case of extremely low sperm/ova number ratios would we expect to see this correlation; exactly this was found in Bjork & Pitnick’s study involving giant sperm *Drosophila* species (Bjork & Pitnick, 2006). In short, male gamete size is typically so small and hence their number so high that further variation in the anisogamy ratio does not result in any detectable signal on the relation between anisogamy and sexual selection. However, aside from this simple “saturation” argument, the intensity of sexual selection could in principle influence the evolution of gamete size under high anisogamy ratios which could make a correlation reappear, and thus we must consider these models too.

We now address these claims in more detail. Why should surplus unfused gametes translate into increased sexual selection intensity on males? There is a very simple and intuitive reason: they generate potential for further fitness gains by achieving fusions with these excess gametes via additional matings (Lehtonen et al., 2016b; Parker, 2014), and this typically results in steeper Bateman gradients for males (the Bateman gradient is the slope of reproductive success on the number of mates (Henshaw & Jones, 2019); see Lehtonen (2022) for a biophysical explanation of the link between gamete numbers and the asymmetry in sex-specific Bateman gradients). We first consider a very simple case, in which both sexes have the same amount of resources for gametes, whatever the degree of anisogamy. Thus, with a size-number trade-off, gamete number ratios remain inversely proportional to gamete size ratios whatever the degree of anisogamy. Under efficient fertilization this potential for further fitness gains saturates quickly (see figures 1–3 in Lehtonen, 2022): once the characteristic Bateman gradient shape emerges, there is little further change with increases in anisogamy. Figure 1 demonstrates this with two simple heuristic models. Equal gametic expenditure is often seen in weakly mobile broadcast spawners, representing an early stage in sexual selection evolution (Parker, 2014). Figure 1A demonstrates how the fraction of unfused male gametes quickly approaches 1, while Figure 1B links gamete numbers to the Bateman gradient in a simple model in which an extra mating can be gained in an initially monogamous population (for details see Supplementary Material 1). Typical metazoan anisogamy ratios, and hence the difference in sperm and ovum numbers, are vastly higher than shown in Figure 1, and therefore for this simple case, we should not expect to see a correlation between anisogamy ratio and sexual selection intensity in most across-species comparisons. Overall, Figure 1 indicates that sexual selection intensity should be associated with anisogamy in a near-binary way, with males typically under stronger sexual selection. Thus here, Claim 1 above is supported by theory but Claim 2 is not.

**Figure 1.**
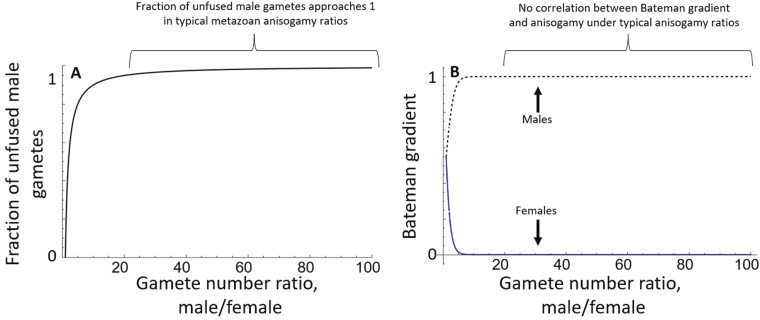
Two very simple heuristic examples of the saturation effect (for further examples, see Lehtonen, 2022; Lehtonen et al., 2016b). Both panels assume a 1:1 sex ratio, and that the number of gametes each female makes is 10, while the number of male gametes per individual varies in the range [10,1000]. When each sex has equal resources for gametes, gamete number ratios match inversely the anisogamy (gamete size) ratios. (A) An example showing the fraction of unused male gametes. Fertilization is assumed to be very efficient, so that all gametes of the less numerous type (female) are fertilized. Since each female makes $n_{x}\;$ = 10 gametes and each male makes $n_{y}$ gametes, the fraction of fused male gametes is thus $n_{x}$ / $n_{y}$ and that of unfused male gametes is 1 − $n_{x}$ / $n_{y}$. With this simple model, across typical anisogamy ratios the unfused fraction would be virtually 1 for males, and 0 for females. This represents untapped reproductive capacity in males which creates the potential for stronger sexual selection in males than in females. (B) A simple model of the Bateman gradient for females (solid lower curve) and males (dashed upper curve), using the Bateman function of Model 1 in Lehtonen (2022) with parameter *a* = 0.1, indicating how much each sex benefits from one additional mating in an initially monogamous population (see Supplementary Material 1). The Bateman gradient for females quickly drops to near zero, while that for males rapidly increases to its maximum value. Here this maximum value is 1 because the gradient has been standardized, but more generally it need not be 1: the crucial point is that in a simple model this measure eventually saturates to some value as the anisogamy ratio increases.

While Figure 1 and the figures in Lehtonen (2022) show simple examples of how sexual selection intensity depends on anisogamy, Lehtonen et al. (2016b) and Figure 1 therein show more specifically how anisogamy causes divergent selection on two sexually competitive traits: selection initially drives males to invest heavily in these traits as the anisogamy ratio increases, but under conditions of efficient fertilization the effect rapidly saturates. To be clear, the issue is not that the two studies (Janicke et al., 2016; Mokos et al., 2021) study organisms in different parts of the curve. Instead, it is an issue of interpretation of (in this regard, admittedly unclear) theory.

In the simple model in Figure 1, we do not explicitly consider any trade-off between investment in preejaculatory sexual competition and male gamete numbers. In many models, resources for reproduction are considered fixed so that preejaculatory sexual competition investment trades off against male gametic expenditure, as has often been assumed and argued theoretically (e.g., Parker (2014); see Lehtonen et al. (2016b) for theoretical framework). Under such a trade-off, in nature a number of selective forces prevent male gamete numbers dropping towards female gamete numbers; that is, sperm limitation, sperm competition and variation in male mating opportunities. Lehtonen et al. (2016b) models a trade-off between gametic investment and investment into sexually selected traits along the isogamy-anisogamy continuum, again finding a saturating effect of anisogamy. In the sexual selection/behavioral ecology tradition a more common way of representing this trade-off in relation to the intensity of sexual selection is by the “time in” ratios of the sexes (Figure 2), that is, a measure of the relative density of males to females that are available for mating. Thus gamete replenishment, parental care, etc. determines “time out” (time unavailable for matings), so that the relative “time in” (time available for acquiring matings) is typically smaller for the sex with more extensive parental care (Clutton-Brock & Parker, 1992); the sex with the greater “time in” therefore experiences more intense sexual selection (Figure 3). This approach is closely related to others such as relative parental investment (Trivers, 1972), operational sex ratio (Emlen & Oring, 1977) and potential reproductive rates (Clutton-Brock & Vincent, 1991). It can also be related to commonly used sexual selection measures based on variance in mating opportunities, which is likely to increase with male time in.

**Figure 2.**
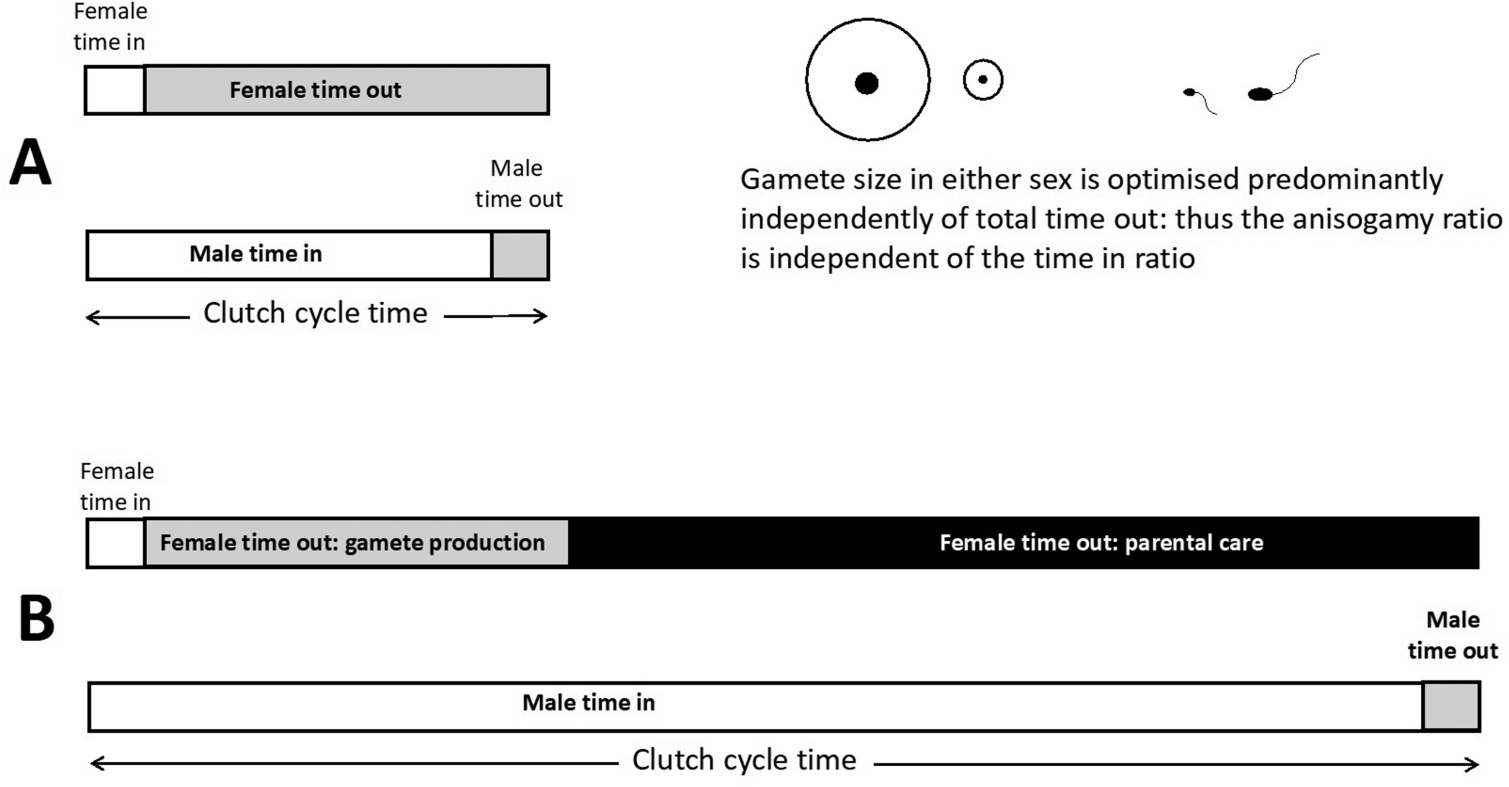
The intensity of sexual selection as represented by the ratio of male/female times in the mating pool (shown as white blocks). This example is constructed with a mobile internal fertilizer in mind, and the adult sex ratio is unity, hence the cycle time (= female time in plus female time out) for both sexes is equal. (A) There is no parental care. Time out of the mating pool (i.e., when an individual is unavailable for matings; gray blocks) is generally high for females, dominated by time gaining the resources required to produce a given clutch. Male time out is generally much less, and includes time gaining resources required to produce the mean number of ejaculates that a female receives per clutch. (B) The effect of adding advanced maternal care (black block). This is likely to dominate female time out, hence greatly increasing the intensity of sexual selection as measured by increase in male time in relative to female time in. Parental care in either sex tends to exert a much greater effect on times out than clutch or ejaculate costs, causing sex role reversal in advanced male-only care.

**Figure 3.**
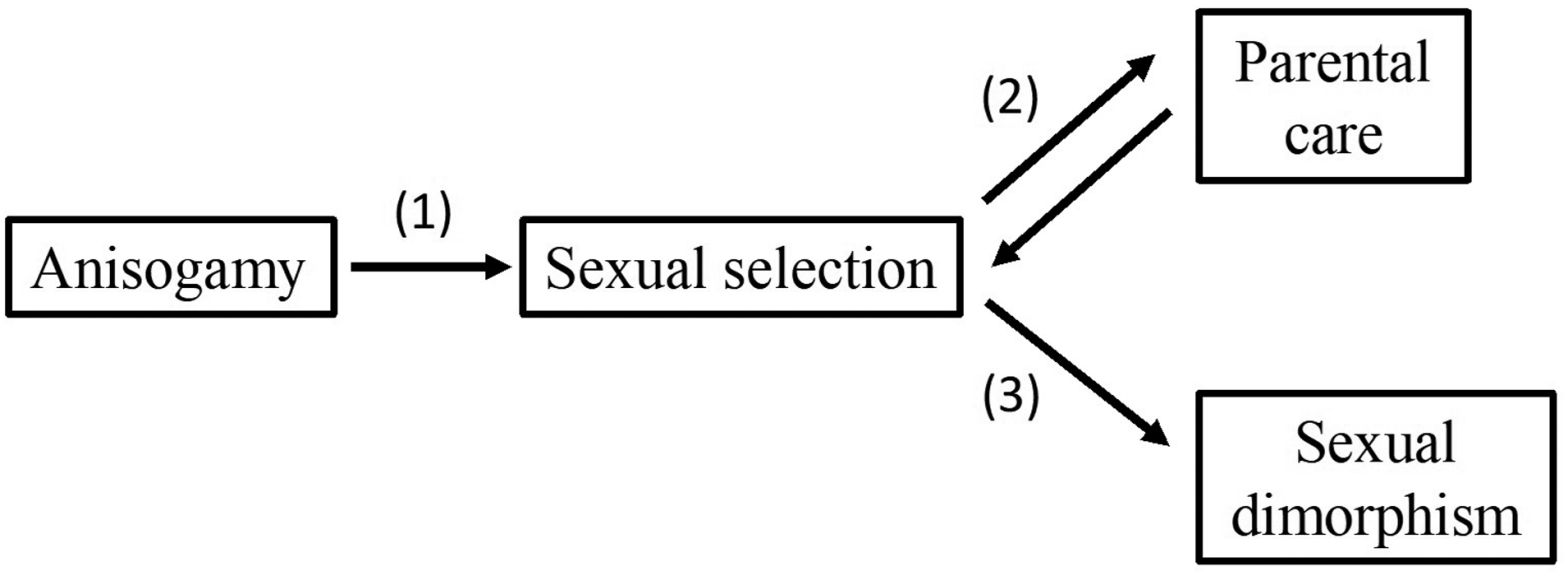
The “Darwin–Bateman paradigm,” after Janicke et al. (2016) and Mokos et al. (2021). While Janicke et al. (2016) found support for all pathways, Mokos et al. (2021) only found support for pathway 2. We have shown that the combined results of both studies are consistent with mathematical predictions.

Can the anisogamy ratio affect the intensity of sexual selection (or vice-versa) under this trade-off? For example, suppose that the optimal sperm size reduces to a half of its former value, doubling the anisogamy ratio. The proportion of unfused gametes then increases. Does this then permit increased expenditure on precopulatory competition, reducing male time out and increasing male time in (Figure 2). Can this increase the intensity of sexual selection? We argue that it will not do so noticeably, for two reasons.

First, even where males become highly mobile and invest less into gametes, their gamete number remains typically vastly greater than females (Lüpold & Fitzpatrick, 2015), maintained by the dual selective forces of sperm competition and sperm limitation (Parker & Lehtonen, 2014). Note that the saturation effect in Figure 1A is not strictly dependent on the assumption of equal gametic expenditures, but only on the divergence in gamete numbers: provided that sperm numbers greatly outweigh ovum numbers, the saturation effect on the Bateman gradients noted in Figure 1B still holds. Consider first the hypothetical case where there is no sperm competition or sperm limitation. At typical high anisogamy ratios, the proportion of unused male gametes approaches 1, so that male time in is near maximal (i.e., approaching the female clutch cycle time). Any increase in the ratio due to the halving of sperm size therefore has an undiscernible effect on the intensity of sexual selection. Because of sperm competition and/or sperm limitation, male gametic investment (and male time out) very rarely approaches zero in nature. These forces increase optimal male gametic expenditure, reducing male time in and the intensity of sexual selection, an effect noted by several authors (e.g., Parker and Birkhead (2013), Lehtonen et al. (2016b)). However, despite this reduction in precopulatory sexual selection intensity, under sperm competition and efficient fertilization, changing the anisogamy ratio across typical ranges again exerts little effect on the (reduced) male gametic investment level (see the lower right panels of Fig. 1 in Lehtonen et al. (2016b)).

Second, there are good reasons to expect that variation in anisogamy ratio due to optimizing the sperm or ovum sizes will not change time in or time out if, plausibly, optimal gamete mass (i.e., gamete size × number) acts as the variable that determines time out cost. Under a size-number trade-off, changes in gamete size will result in inverse changes in gamete number, so that for a given amount of resources available for reproduction, their product (gamete mass) remains constant. At high anisogamy ratios, sperm make no significant provisioning contribution to the zygote (Parker, 1982) and ovum size is determined by highly variable selective forces across species relating to zygote survival (e.g., Parker & Begon, 1986; Smith & Fretwell, 1974), generating a wide range of ovum sizes. However, if clutch mass is optimized, then at least simplistically, increasing ovum size decreases ovum number but does not alter optimal clutch mass, hence female time out remains unchanged. Similarly, sperm size is also variable depending on phylogeny and fertilization ecology, and is typically tiny compared to the ovum, but optimized quite differently, in relation to maximizing fertilization success (e.g., Immler et al., 2011; Parker, 1993; Parker et al., 2010). Again, under the simplest assumptions, sperm size is optimized after Smith-Fretwell principles (1974): in simple sperm competition game models it can be shown that the product of sperm size and number act as a single variable (see Supplementary Material 2). Thus, changing the optimal sperm size changes the sperm number, but not male gametic expenditure, or therefore, male time out. In either sex, adding the dominating effect of parental care can nevertheless notably affect the asymmetry, as shown in Figure 2.

Hence although anisogamy initially determines the direction of sexual selection, existing theory using Bateman gradients suggests that the effect saturates quickly (following Figure 1) thus predicting that any further signal quickly disappears. Under advanced anisogamy, models that directly link anisogamy (or the closely associated gamete number) to sexual selection (Lehtonen et al., 2016b; Lehtonen, 2022, and Figure 1 above) predict exactly what the combined results of Janicke et al. (2016) and Mokos et al. (2021) show: a difference in gamete size and number causes stronger sexual selection in males, but that sexual selection intensity is not obviously correlated with the anisogamy ratio in typical anisogamous organisms.

Let us recapitulate all the pathways of the “Darwin-Bateman paradigm” studied by Janicke et al. (2016) and Mokos et al. (2021) (Figure 3) alongside the relevant theoretical predictions.

Pathway 1: the theoretical prediction regarding anisogamy is that it is centrally important as a qualitative predictor of sexual selection intensity, but, models linking anisogamy or gamete numbers directly to sexual selection predict a near-binary relationship: sexual selection should be on average stronger in microgamete producers (males), but across organisms with typical gamete size and number ratios, the correlation between the degree of anisogamy and sexual selection intensity is predicted to be so weak as to be undetectable, exactly as the two seemingly contradictory studies of Janicke et al. (2016) and Mokos et al. (2021) found. Note that in Figure 3 an arrow flows from anisogamy to sexual selection, but not in the opposite direction. Theory supports this: although anisogamy sets an ancestral binary bias in sexual selection direction that persists across most species [Janicke et al. (2016)], gamete sizes are determined by factors largely unrelated to its intensity, as Mokos et al. (2021) found. Thus, while both studies are valuable for our understanding of sexual selection, neither causes any controversy with the Darwin-Bateman paradigm, though they may appear to do so at first sight. When current theory is carefully examined, the prediction that Mokos et al. (2021) appear to falsify turns out to not be a prediction of sexual selection theory; rather, their study turns out to fit theoretical predictions.

Pathway 2: Both Janicke et al. (2016) and Mokos et al. (2021) found that parental care was clearly associated with sexual selection intensity, which was greater in males of species with female-biased parental care. This is again consistent with previous theory on sexual selection intensity: if parental care is provided only by females, we might expect sexual selection to be more intense in males (Figure 2B), and vice-versa. Furthermore, recent theory shows that causality goes both ways: the sex under more intense sexual selection is expected to evolve a lower level of parental care (Fromhage & Jennions, 2016). Both predictions are consistent with Janicke et al. (2016) and Mokos et al. (2021).

Regardless of the absence/presence of parental care, unless special conditions apply, the optimal gamete sizes in either males or females are independent of that sex’s time out of the mating pool, typical ovum/sperm size ratios are therefore unrelated to male/female times out ratios (Figure 2).

Pathway 3: Both studies also tested associations between various forms of sexual dimorphism and sexual selection intensity, with only one (Janicke et al., 2016) finding a clear association. However, their results are not directly comparable: one (Mokos et al., 2021) specifically tested for an association between size dimorphism and sexual selection, while the other (Janicke et al., 2016) explicitly excluded size dimorphism. It is therefore perhaps unsurprising that the latter found an association between their measure of sexual dimorphism and sexual selection, while the former did not: sexual selection theory certainly predicts sexual dimorphism, but male body size may either decrease (e.g., if males compete by agility) or increase (e.g., if they compete by combat) (Mokos et al., 2021).

A summary of how gamete size evolution is expected to influence sexual selection is given in Table 1. We see anisogamy as having had a notable and lasting qualitative effect on sexual selection through its evolutionary history, but it is anisogamy per se, not its quantitative degree, that generates the widespread difference in sexual selection between the sexes. Our view is that while anisogamy has generated what may be termed an “ancestral mainstream binary division of sex roles,” as detected by Janicke et al. (2016), it is, however, not anisogamy, but ecological, sociobiological and other factors that secondarily modify and occasionally even reverse the sex-specific intensity of sexual selection, for example, under secondary adaptations such as paternal care. Mokos et al. (2021) offer valuable support for this “binary” view. Thus, the important studies of Janicke et al. and Mokos et al. both shed light on the nature of sexual selection when interpreted in the light of sexual selection theory.

**Table 1.** The big picture of how gamete size evolution influences sexual selection.

	Ancestral isogamy	Very low anisogamy ratios (low female/male gamete masses)	Typical very high anisogamy ratios (high female/male gamete masses)	Organisms with parental care	Overall broad-scale predictions
Predicted effect of sexual selection	No consistent difference in intensity of sexual selection between mating types.	Divergence of gamete sizes and numbers provides initial impetus for mainstream flow of sexual differentiation. Theory predicts a clear correlation between anisogamy ratio and strength of precopulatory sexual selection only when anisogamy ratio is very low.	No predicted overall correlation between anisogamy ratio and intensity of precopulatory sexual selection, because the direct effect of gamete number asymmetry saturates quickly and also gamete sizes typically optimized independently of sexual selection under size/number trade-off.	Higher intensity of sexual selection likely on the sex with less extensive parental care.	1) The isogamy-anisogamy transition causes sexual selection such that on average, sexual selection is stronger on the microgamete producer. 2) A correlation between anisogamy ratio and intensity of precopulatory sexual selection is *not expected* under typical anisogamy ratios. 3) A correlation between the extent of parental care and the intensity of sexual selection *is expected*, such that precopulatory sexual selection is generally stronger in the sex with less extensive parental care.
Biological occurrence	Found in many unicellular organisms and some multicellular algae (e.g., Lehtonen et al., 2016a).	Such low anisogamy ratios very rare in Metazoa (e.g., a few *Drosophila* species: Bjork & Pitnick, 2006); found in some algae (e.g., *Monostroma angicava*: Togashi et al., 2015).	Found in almost all Metazoa, vascular plants and most multicellular algae (e.g., Lehtonen et al., 2016a)	Found in Metazoa, several taxa show maternal care, some show paternal care.	General conformance with empirical findings: mobile anisogamous metazoans typically show more intense precopulatory sexual selection in the microgamete producer, i.e., males [Janicke et al., (2016)], but beyond this binary relationship there is no sustained relation between anisogamy ratio and intensity of precopulatory sexual selection across typical anisogamy ratios [Mokos et al., (2021)].
					Sexual selection has been found to be more intense on the sex providing less parental care (Mokos et al., 2021).

## Supplementary material

Supplementary material is available online at *Evolution Letters*.

qrae029_suppl_Supplementary_Material

## Data Availability

No new data were generated or analyzed in support of this research.
