# Supplementary material for: The correlation between anisogamy and sexual selection intensity—the broad theoretical predictions
Source: Evol Lett. 2024 Jul 12;8(6):749–55. doi: 10.1093/evlett/qrae029 (PMC11637515; doi:10.1093/evlett/qrae029)
Supplement: qrae029_suppl_Supplementary_Material [file qrae029_suppl_supplementary_material.pdf]

Supplementary Information for: The correlation between anisogamy and sexual selection intensity – the broad theoretical predictions

Jussi Lehtonen, Department of Biological and Environmental Science, University of Jyväskylä, 40014 Jyväskylä, Finland

Geoff Parker, Department of Evolution, Ecology and Behaviour, University of Liverpool, Liverpool, L69 7ZB, UK.

SI.1 Calculation of Bateman gradients in Fig. 1b

The Bateman gradient in Fig. 1b is computed directly using the Bateman function of Model 1 in Lehtonen (2022),

$$b_x(p) = f(n_x, pn_y) \qquad \text{(S1.1)}$$

where in an initially monogamous population a mutant individual of type x (producing n_x gametes) is able to attract p fertilisation partners of type y (producing n_y gametes). The function f is a 'fertilisation function' (Lehtonen and Dardare 2019) $f(N_x, N_y) = N_x N_y \frac{e^{aN_x} - e^{aN_y}}{N_x e^{aN_x} - N_y e^{aN_y}}$, where N_x and N_y are total gamete numbers of each type in the fertilisation arena and a is a parameter controlling fertilisation efficiency (for the special case $N_x = N_y$ the function is defined as $f(N_x, N_y) = \frac{aN_x^2}{1+aN_x}$ (Lehtonen and Dardare 2019), which is also the limit of f when $N_y \to N_x$). In Fig. 1b we use the value $a = 0.1$ which implies reasonably efficient fertilisation.

The Bateman gradient is calculated simply as the difference in the value of the above Bateman function when the mutant individual mates twice versus monogamy, standardised by dividing by the mean (resident) value:

$$B = \frac{b_x(2) - b_x(1)}{b_x(1)} \qquad \text{(S1.2).}$$

We then fix the number of 'female' gametes to $n_x = 10$, and calculate the value of the Bateman gradient for both sexes when n_y varies from 10 to 1000. The result is Figure 1b where the female Bateman gradient is shown in Blue and the male Bateman gradient in black.

Analogous results saturating in a similar way can be derived using the Bateman functions of Model 2 and 3 in (Lehtonen 2022).

References

Lehtonen, J. 2022. Bateman gradients from first principles. Nature Communications 13:3591.

Lehtonen, J. and L. Dardare. 2019. Mathematical Models of Fertilization—An Eco-Evolutionary Perspective. The Quarterly Review of Biology 94:177-208.

SI. 2 Should anisogamy ratio relate to pre-copulatory sexual competition under strong anisogamy ?

The 'time in – time out' model (Fig. 2) suggests that as male ejaculate investment increases, the intensity of sexual selection will decrease because male time in decreases. Here we show that although male ejaculate and/or female clutch expenditures will affect sexual selection, these will not, at least on simple assumptions, relate to the degree of anisogamy.

First consider sperm size. Ejaculate investment (relating to male time out in Fig. 2) is increased (and hence pre-copulatory sexual selection intensity on males, relating to time in, Fig. 2, is reduced) by sperm competition and sperm limitation (see e.g., Parker et al. 2018). Here we demonstrate in a simple model that though ejaculate mass (male post-copulatory expenditure) increases with sperm competition level, the ESS sperm size can change independently of sperm competition level, so that changes the anisogamy ratio due to changes in ESS sperm size should be unrelated to pre-copulatory sexual selection intensity). Similarly, we argue that when female clutch size is increased, though this may increase female time out, and hence cycle length, thus increasing male time in (see Fig. 2) – this (again on simple assumptions) can occur independently of the degree of anisogamy.

We examine the typical case of strong anisogamy, where the sperm does not contribute significantly to the provisioning of the zygote, a fundamental assumption of 'sperm competition games' (SCG) (for justification, see Parker 1982, Lehtonen & Kokko 2011). The following analysis represents a merger of the derivation of ESS sperm size in the 'risk model' of sperm competition by Parker (1993) with a modified version of the time in - time out model for ESS sperm allocation under the 'intensity model' by Parker & Ball (2005). SCGs assume a trade-off between pre-and post-ejaculatory components of male-male competition, and use an ESS approach to deduce the balance between these components. In the simplest 'mate search competition' SCG version, the total resource budget for reproduction is R, C is the search cost (in the same units) of finding a receptive female, m is the size of each sperm, and s is the number of sperm in each ejaculate. Where D is the cost of each unit of ejaculate, the total ejaculate cost is Dsm. Hence the ejaculate cost as a proportion of the expenditure on a given mating is $Dsm/(C + Dsm)$, and the relative total number of matings per male is $R/(C + Dsm)$. The relative fertilising ability of a sperm of size m is defined by the function $g(m)$.

If the female accepts P_f matings per cycle, it follows that the average number of matings per male per female cycle is $P_m = P_f/\beta$, where β is the adult sex ratio (males/females). For simplicity we shall here assume that $\beta = 1$, so that $P_m = P_f = P$, which is a measure of the intensity of sperm competition. Where k is a positive constant, we can write

$$P = kR/(C + Dsm). \qquad \text{(S2.1)}$$

While the population level of P is set by the female, a mutant male can alter his number of matings, p, by varying his ejaculate expenditure. Using $\hat{s}$, $\hat{m}$ to denote population strategies and $s \neq \hat{s}, m \neq \hat{m}$ for mutant strategies, the number of matings gained by a mutant male is

$$p(s, \hat{s}) = kR/(C + Ds\hat{m}), \qquad \text{(S2.2a)}$$

$$p(m, \hat{m}) = kR/(C + D\hat{s}m). \qquad \text{(S2.2b)}$$

Thus the value of a mating to a mutant male playing s is therefore

$$v(s,\hat{s},\hat{m}) = \left(\frac{sg(\hat{m})}{(P-1)\hat{s}g(\hat{m})+sg(\hat{m})}\right) = \left(\frac{s}{(P-1)\hat{s}+s}\right). \quad \text{(S2.3a)}$$

For a mutant playing m

$$v(m,\hat{m},\hat{s}) = \left(\frac{\hat{s}g(m)}{(P-1)\hat{s}g(\hat{m})+\hat{s}g(m)}\right) = \left(\frac{g(m)}{(P-1)g(\hat{m})+g(m)}\right). \quad \text{(S2.3b)}$$

Calling mutant fitness $w = pv$, we can obtain the ESS values, s^*, m^* in the usual way by setting

$$\left.\frac{dw}{ds}\right]_{s=s^*} = 0, \qquad \left.\frac{dw}{dm}\right]_{m=m^*} = 0,$$

which gives for each mutant, when $s = s^*, m = m^*$,

$$\frac{p'}{p} = -\frac{v'}{v}\ , \quad \text{(S2.4)}$$

(see Parker 1993). Since at the ESS, $p = P$, for an s mutant we obtain

$$\frac{p'}{p} = \frac{(0-kRDm^*)}{(C+Ds^*m^*)^2}\frac{(C+Ds^*m^*)}{kR} = -\frac{Dm^*}{(C+Ds^*m^*)}, \quad \text{(S2.5)}$$

$$-\frac{v'}{v} = -\frac{(Ps^*-s^*)}{(Ps^*)^2}\frac{P^*}{s^*} = -\frac{(P-1)}{Ps^*}, \quad \text{(S2.6)}$$

and inserting (S2.5) and (S2.6) into (S2.4) gives

$$\frac{Ds^*m^*}{(C+Ds^*m^*)} = \frac{(P-1)}{P}, \quad \text{(S2.7)}$$

see Parker & Ball (2005), equation (2.11b) and in earlier papers, which stress that the proportion of reproductive resources spent on the ejaculate should increase with the intensity of sperm competition. Note that ejaculate expenditure s^*m^* acts as a single variable that is optimised in (S2.7) since all other terms can be regarded as constant, thus a given increase in sperm size m^* generates a proportionate decrease in sperm number s^*.

Similarly, we derive the ESS for sperm size, m^*, from (S2.4)

$$\frac{p'}{p} = \frac{(0-RDs^*)}{(C+Ds^*m^*)^2}\frac{(C+Ds^*m^*)}{R} = -\frac{Ds^*}{(C+Ds^*m^*)}, \quad \text{(S2.8)}$$

$$-\frac{v'}{v} = -\frac{(g'Pg-g'g)}{(Pg)^2}\frac{Pg}{g} = -\frac{g'(P-1)}{Pg}, \quad \text{(S2.9)}$$

hence applying (S2.4) we get

$$\frac{Ds^*}{(C+Ds^*m^*)} = \frac{g'(P-1)}{Pg}\ . \quad \text{(S2.10)}$$

From (S2.7) and (S2.10) we then see that

$$\frac{Ds^*}{(C+Ds^*m^*)} = \frac{g'}{g}\left(\frac{Ds^*m^*}{(C+Ds^*m^*)}\right),$$

hence

$$g'(m^*) = \frac{g(m^*)}{m^*} \quad \text{(S2.11)}$$

which is the familiar Smith-Fretwell (1974) marginal value for solution for propagule size and can be satisfied if the function $g(m)$ has a suitable increasing form (see Parker 1993 for a similar result in an early version of the sperm competition risk model).

Note that in this simple case, the relative expenditure on the ejaculate (Ds^*m^*) increases with sperm competition intensity. However, ESS sperm size, m^*, is unrelated to the level of sperm competition, and hence unrelated to the relative levels of pre- versus post-copulatory expenditure. More complex analyses of sperm size (see Parker et al. 2010) show that m^* can relate to sperm competition level P if function $g(m)$ varies with sperm density in the female tract (or the fertilization arena if there is external fertilization).

And ovum size? Provided that anisogamy has reached a typically high level, sperm function $g(m)$ is optimised for fertilization success, and not for contributing resources to the zygote: the zygote becomes provisioned by the female gamete (Parker 1982, Lehtonen & Kokko 2011). The ovum mass is then optimised independently of sperm size, again most simplistically following the Smith-Fretwell solution derived above in (S2.11) (Parker 1982, 2011). Thus male gamete size, m_y^* typically finds a minimal level set by fertilization success, and female gamete size, m_x^*, will be set by phylogeny and the ecology of reproduction in given taxa, and is highly diverse. Obviously whatever reserves are left in the sperm at fusion can be used by the zygote – but this is only likely to be significant in weakly anisogamous species, in which the male gamete may contribute to zygote provisioning. However, only a tiny proportion of metazoans can be considered to be weakly anisogamous (e.g. species with giant sperm).

So once anisogamy has proceeded to a significant level, sperm size and ovum size are subject to radically different selective forces; on the simplest assumptions they are optimized independently and unlikely to correlate with the intensity of sexual selection: sexual selection does not influence gamete size, and gamete size does not influence sexual selection.

How does this relate to the time in – time out model of sexual selection intensity (Fig. 2)? Here, time in (proportional to PC) is set by both the female cycle duration and the male time out (proportional to PDs^*m^*). Calling the female cycle duration T, we expect this to equal the sum of male times in and out; i.e. when β (adult sex ratio) = 1

$$T \propto P(C + Ds^*m^*),$$

so multiplying both sides of (S2.7) by $P(C + Ds^*m^*)$, the sum of ejaculate expenditures is

$$PDs^*m^* \propto \frac{T(P-1)}{P}. \quad \text{(S2.12)}$$

So again, although PDs^*m^* increases as expected with T and P, at any given values of T and P changes in sperm size m^* (and hence anisogamy ratio) will not affect PDs^*m^*, which remains constant.

Thus once the anisogamy ratio has achieved its typically high level, this simple example indicates why it is not unexpected that there is no relationship between anisogamy ratio and the intensity of pre-copulatory sexual selection on males.

References

Lehtonen, J. and H. Kokko. 2011. Two roads to two sexes: unifying gamete competition and gamete limitation in a single model of anisogamy evolution. Behavioral Ecology and Sociobiology 65:445-459.

Parker, G. A. 1982. Why are there so many tiny sperm? Sperm competition and the maintenance of two sexes. Journal of Theoretical Biology 96:281-294.

Parker, G. A. 1993. Sperm competition games: sperm size and sperm number under adult control. Proceedings of the Royal Society of London B: Biological Sciences 253:245-254.

Parker, G. A. & Ball, M. A. 2005. Sperm competition, mating rate and the evolution of testis and ejaculate sizes: a population model. Biology Letters 1, 235-238.

Parker, G. A. 2011. The origin and maintenance of two sexes (anisogamy), and their gamete sizes by gamete competition. Pp. 17-74 *in* T. Togashi, and P. A. Cox, eds. The evolution of anisogamy: a fundamental phenomenon underlying sexual selection Cambridge University Press, Cambridge.

Parker, G. A., S. Immler, S. Pitnick, and T. Birkhead. 2010. Sperm competition games: Sperm size (mass) and number under raffle and displacement, and the evolution of P2. Journal of Theoretical Biology 264:1003-1023.

Parker, G. A., S. A. Ramm, J. Lehtonen, and J. M. Henshaw. 2018. The evolution of gonad expenditure and gonadosomatic index (GSI) in male and female broadcast-spawning invertebrates. Biological Reviews 93:693-753.

Smith, C. C. and S. D. Fretwell. 1974. The optimal balance between size and number of offspring. The American Naturalist 108:499-506.
